# Medida da rotação interna do joelho no diagnóstico clínico da associação de lesão no ligamento anterolateral e no ligamento cruzado anterior

**DOI:** 10.1055/s-0045-1811633

**Published:** 2025-11-04

**Authors:** Geraldo Luiz Schuck de Freitas, João Luiz Ellera Gomes

**Affiliations:** 1 Complexo Hospitalar da Santa Casa de Porto Alegre, Universidade Federal do Rio Grande do Sul, Porto Alegre, RS, Brasil; 2 Universidade Federal do Rio Grande do Sul, Porto Alegre, RS, Brasil; 3 Serviço de Ortopedia e Traumatologia do Hospital de Clínicas de Porto Alegre, Porto Alegre, RS, Brasil

**Keywords:** amplitude de movimento articular, articulação do joelho, diagnóstico, ligamento cruzado anterior, anterior cruciate ligament, diagnosis, knee joint, range of motion, articular

## Abstract

**Objetivo:**

Investigar a correlação clínica entre a rotação interna do joelho e a associação de lesões nos ligamentos anterolateral (LAL) e cruzado anterior (LCA).

**Métodos:**

Trinta e oito joelhos de 19 cadáveres frescos (todos do sexo masculino, média de idade: 28 anos) foram avaliados simulando o exame físico por meio de testes manuais de rotação em 90 graus de flexão. Fios de Kirschner foram colocados paralelamente no fêmur e na tíbia e as medidas foram obtidas com um goniômetro. Os dados obtidos foram comparados com o LCA intacto e, em seguida, com secções progressivas do LCA, trato iliotibial (TIT) e LAL.

**Resultados:**

A secção isolada do LCA induziu um aumento (+55,6%, *p* < 0,001) na rotação interna em 90 graus de flexão em comparação a de o LCA intacto. Após a secção do LCA, a liberação associada do TIT induziu um aumento (+31,6%, *p* < 0,001) na rotação interna do joelho em 90 graus de flexão e um aumento acentuado (+104%, *p* < 0,001) em comparação ao joelho com LCA intacto. Após a secção do LCA e do TIT, a liberação do LAL induziu aumentos significativos (+27,8%, *p* < 0,001) na rotação interna do joelho em 90 graus de flexão e em comparação ao joelho com LCA intacto (+162%, *p* < 0,001).

**Conclusão:**

Há um aumento da rotação interna do joelho na lesão do LCA. A associação com a lesão do LAL causa um aumento pronunciado da rotação interna em comparação ao joelho íntegro. Portanto, a presença de rotação interna pronunciada do joelho é um sinal clínico de lesão associada dessas estruturas.

## Abstract (en)

**Objective:**

To investigate the clinical correlation between internal knee rotation and the association of injuries between anterolateral (ALL) and anterior cruciate (ACL) ligament.

**Methods:**

Thirty-eight knees of 19 fresh corpses (all males, mean age: 28-years-old) were evaluated by simulating physical examination through manual rotational tests at 90 degrees of flexion. Kirschner wires were placed in parallel in the femur and tibia, and measurements were obtained using a goniometer. The obtained data were compared against the intact ACL, then to progressive sections of the ACL, iliotibial tract, and anterolateral ligament.

**Results:**

Isolated release of the ACL induced an increase (+55.6%, *p* < 0.001) in internal rotation at 90 degrees of flexion, when compared to the internal rotation of the intact knee. After ACL release, associated release of the iliotibial tract (ITT) induced an increase (+31.6%, *p* < 0.001) in the internal rotation of the knee at 90 degrees of flexion, and a marked increase (+104%, *p* < 0.001) when compared to the ACL-intact knee. After ACL and ITT release, an additional ALL release induced a significant increase (+27.8%, *p* < 0.001) in the internal rotation of the knee at 90 degrees of flexion, and in comparison with the intact ACL knee (+162%, *p* < 0.001).

**Conclusion:**

There is an increase in internal rotation of the knee in the ACL injury. The association with ALL injury leads to a pronounced increase of internal rotation when compared to the uninjured knee. Therefore, the presence of pronounced internal knee rotation is a clinical sign of associated injury to these structures.

## Introdução

Estudos sobre a existência de uma estrutura ligamentar distinta na face anterolateral do joelho (LAL), 1 2 reacenderam a discussão sobre a instabilidade de rotação do joelho após a lesão do ligamento cruzado anterior (LCA). Em joelhos lesionados, a instabilidade provoca anteriorização da tíbia no plano anteroposterior e aumento da rotação interna do joelho.

Uma importante correlação entre a instabilidade rotacional e lesões de estruturas anterolaterais é evidente. 3 No entanto, ainda não se sabe qual estrutura tem papel mais importante. Historicamente, os cirurgiões consideram que o controle de rotação da tíbia assegura a estabilidade do joelho. 4

Até o momento, porém, não há consenso sobre qual procedimento gera o melhor controle de rotação na reconstrução do LCA. 5 6 Revisões sistemáticas recentes concluíram que, em alguns casos, a combinação de reconstruções intra- e extra-articulares do LCA poderia melhorar a instabilidade da rotação. 7 8 A existência de um sinal clínico que pudesse melhorar a identificação da associação de lesões do LCA e do LAL ajudaria a identificação dos pacientes que se beneficiariam de reconstrução combinada.

Este estudo teve como objetivo determinar a medida da rotação interna do joelho que pudesse demonstrar claramente a associação entre o LCA e o LAL. Mais especificamente, esta investigação se concentrou na avaliação clínica para auxiliar os cirurgiões no diagnóstico dessas lesões associadas. Nossa hipótese era a possibilidade de definição clínica de uma lesão associada das estruturas anterolaterais do joelho, com insuficiência do LCA por meio da medida da rotação interna.

## Materiais e Métodos

Um total de 19 cadáveres inteiros foram selecionados, compreendendo 38 joelhos sem evidência de lesão ligamentar, condral, ou meniscal, sendo a amplitude de movimento mínima de 0 a 130 graus. Todos os cadáveres foram obtidos no Instituto Médico Legal local de acordo com o protocolo aprovado pelo Comitê de Ética, sob o CAAE: 45087815.0.0000.5327. A idade média dos doadores foi de 28,42 anos (variação: 18–47). Todos os espécimes eram frescos, com menos de 18 horas após a morte, e nenhum havia sido previamente congelado.

### Abordagem Cirúrgica

**Fig. 1:**
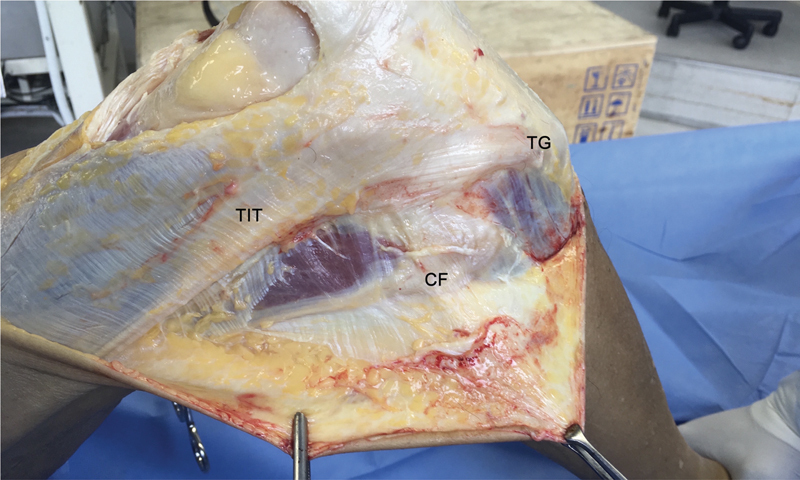
Vista da face lateral do joelho direito após a remoção da pele nas superfícies anteriores e anterolaterais do joelho, criando uma grande janela retangular. O trato iliotibial (TIT) foi, então, identificado. **Abreviações:** CF, cabeça da fíbula; TG, tubérculo de Gerdy.

A dissecção de 38 joelhos de 19 cadáveres frescos utilizou um protocolo padronizado 9 para abordagem do LCA, do trato iliotibial (TIT) e do LAL de ambos os joelhos. Antes do experimento, realizamos a dissecção anatômica adequada das estruturas, começando com a remoção da pele das faces anteriores e anterolaterais do joelho, criando uma grande janela retangular. O TIT foi identificado ( Fig. 1 ) e uma artrotomia parapatelar medial liberou o tendão do quadríceps para expor a região intercondilar e o LCA. O viés produzido pela rigidez cadavérica foi evitado, uma vez que nem todos os espécimes tinham exatamente do mesmo tempo post mortem.

O membro inferior foi posicionado com o quadril flexionado a 45 graus, o joelho flexionado a 90 graus e o pé apoiado na mesa. Após a artrotomia parapatelar medial para identificação da região intercondilar femoral e das estruturas de interesse, antes do início do experimento, todos os espécimes foram avaliados quanto à presença de alguma lesão. Em seguida, com os joelhos posicionados em 90 graus de flexão, dois fios de Kirschner 2.0 paralelos foram inseridos, um no teto intercondilar femoral e o outro na tuberosidade anterior da tíbia.

**Fig. 2:**
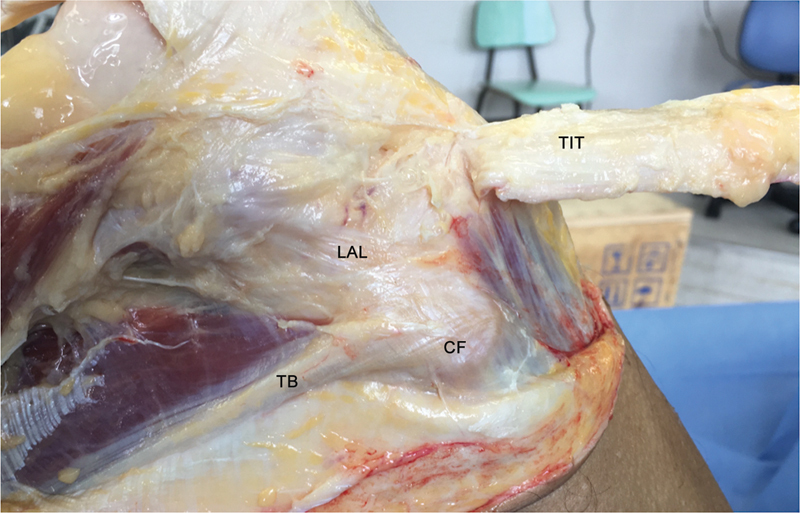
Vista lateral do joelho direito. O trato iliotibial (TIT) é dissecado e rebatido anteriormente para expor o ligamento anterolateral (LAL). Isso permitiu o acesso ao LAL para sua identificação e secção durante o experimento. **Abreviações:** CF, cabeça da fíbula; TB, tendão do bíceps.

A abordagem foi estendida lateralmente, começando sobre o tubérculo de Gerdy e seguindo em sentido proximal sobre a coxa por 30 cm. Em seguida, foi realizada a liberação sequencial do LCA e das estruturas anterolaterais do joelho, iniciando no TIT e avançando para o LAL, conforme o protocolo experimental ( Fig. 2 ).

### Experimento

**Fig. 3:**
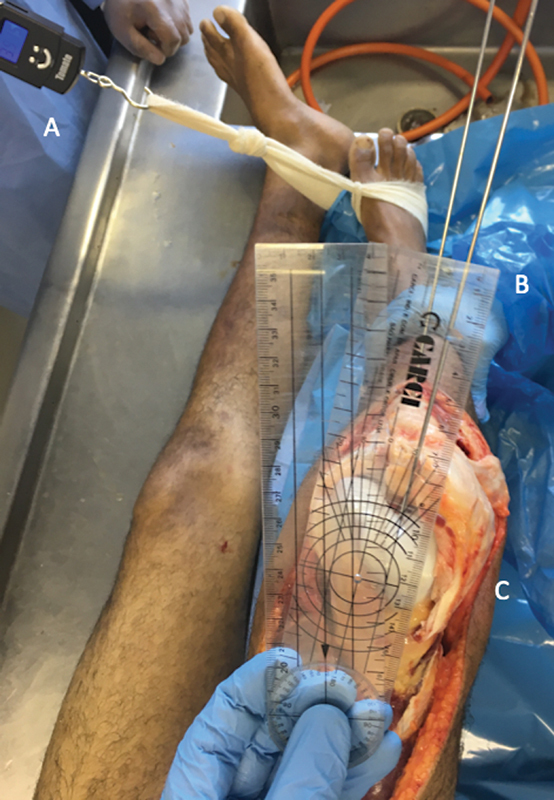
Vista anterior do joelho direito, mostrando os fios de Kirschner posicionados, ( **B** ) um no teto do intercôndilo femoral e outro na tuberosidade anterior da tíbia. A rotação interna máxima foi ( **A** ) realizada com um dinamômetro e ( **C** ) medida com um goniômetro.

Os dados cinemáticos foram obtidos com um goniômetro, que mediu o ângulo formado entre os dois fios de Kirschner previamente posicionados. No início do experimento, antes da secção ligamentar, determinamos a rotação interna máxima da tíbia em relação ao fêmur fixo a 90 graus de flexão. Um dinamômetro STC-02 (Mundial Comércio de Presentes Ltda.) foi utilizado para obter um padrão de força durante a rotação. Aplicamos tração máxima até o ponto em que a rotação foi contida pela ação ligamentar no joelho normal, antes das secções. Em seguida, registramos a leitura do dinamômetro para determinar a força máxima que poderia ser aplicada àquele espécime pelo restante do experimento. A força de rotação foi aplicada após cada liberação até a obtenção do mesmo valor que o joelho normal no dinamômetro. Isto controlou o viés de avaliação ( Fig. 3 ). As medidas foram obtidas assim que a rotação máxima foi atingida.

**Fig. 4:**
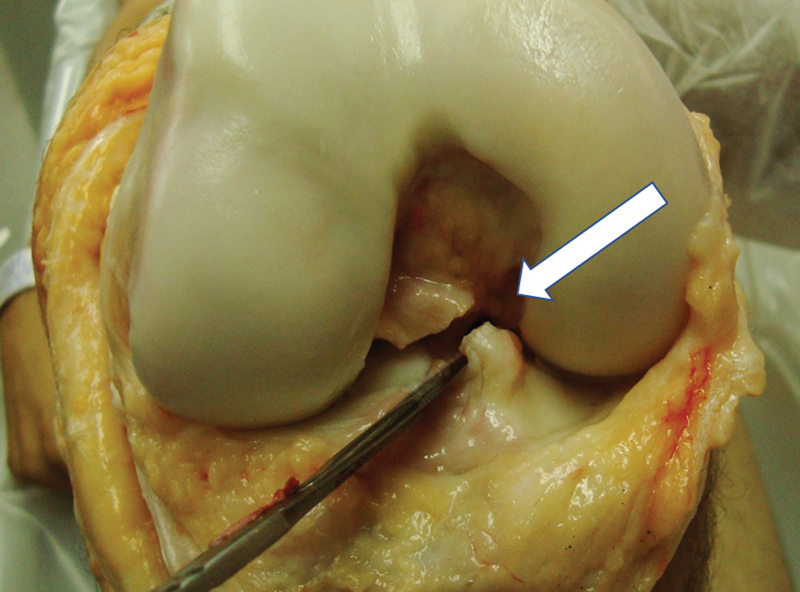
Vista anterior do joelho direito em 90 graus de flexão. A seta branca mostra a secção do LCA com o bisturi. A seguir, a rotação interna da tíbia foi medida.

Com o joelho mantido a 90 graus, atingimos a rotação interna máxima e medimos o ângulo entre os dois fios de Kirschner com um goniômetro (PVC, 35 cm). Esses dados foram coletados em um grupo denominado “ligamento cruzado anterior intacto” (LCAI). Dando continuidade ao experimento, o LCA foi seccionado cirurgicamente ( Fig. 4 ) e as mesmas medidas foram obtidas e coletadas em um grupo denominado ligamento cruzado anterior com lesão (LCA L).

No estágio seguinte do experimento, o TIT foi submetido à secção cirúrgica proximal e refletido inferiormente ( Fig. 2 ). Os pesquisadores tiveram cuidado para não atingir seu sítio de inserção na tíbia.

**Fig. 5:**
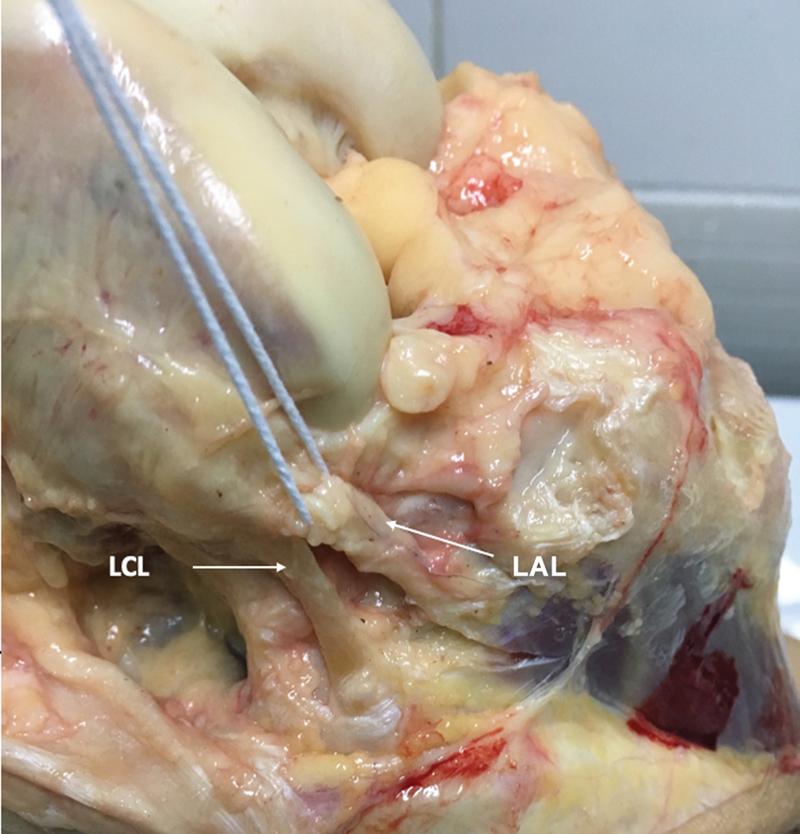
Vista lateral do joelho direito após a dissecção, rebatimento anterior e remoção do trato iliotibial. O ligamento anterolateral (LAL) pode ser individualizado e dissecado, separando-o do ligamento cruzado lateral (LCL). Em seguida, o LAL foi seccionado e procedemos à mensuração da rotação interna da tíbia.

As medidas foram obtidas com a mesma técnica já descrita e os dados foram coletados em um grupo denominado ligamento cruzado anterior com lesão e TIT com lesão (LCA L + TIT). Nesta etapa do procedimento, após a retração do TIT, o LAL foi dissecado, aplicando-se força de varo e rotação interna em 30 e 60 graus de flexão para elicitar o efeito dessas estruturas como o LAL, sob tensão, descrito como a resistência ao movimento, 4 como pode ser visto na Fig. 5 .

**Fig. 6:**
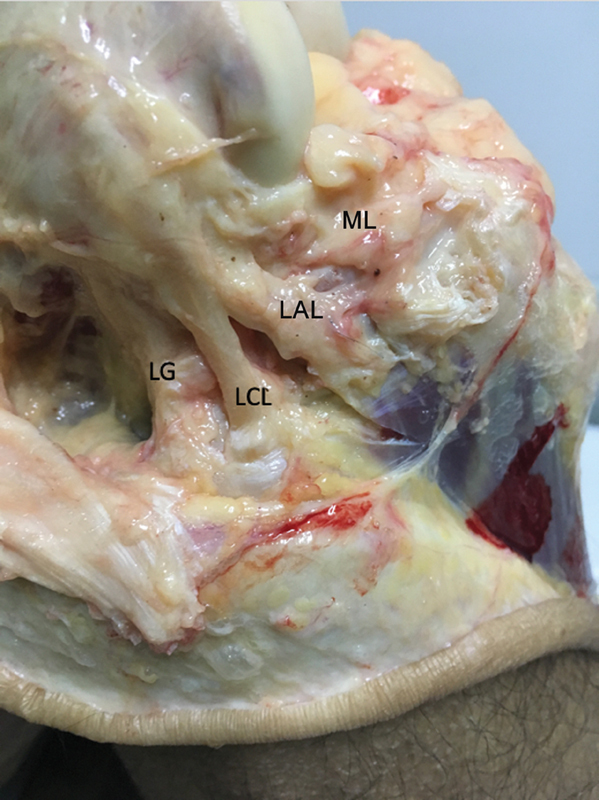
Vista lateral do joelho direito mostrando as estruturas laterais da articulação. De trás para a frente, cabeça do gastrocnêmio lateral (LG), ligamento colateral lateral (LCL), ligamento anterolateral (LAL) e menisco lateral (ML). O tendão poplíteo não pode ser visualizado, pois fica abaixo do LCL e do LAL.

Após a exposição da área de interesse contendo o LAL, o ligamento colateral lateral (LCL) e o tendão poplíteo (TP) foram identificados ( Fig. 6 ). O LCL foi identificado por palpação de sua estrutura cilíndrica no sítio de inserção distal à cabeça da fíbula, logo acima da inserção do tendão do bíceps femoral. Em seguida, foi exposto em sentido posterior para evitar a ruptura de qualquer tecido no aspecto anterolateral. Para confirmar que nenhuma parte do LCL havia sido confundida com qualquer outra estrutura, ele foi completamente isolado de todas as outras estruturas circundantes, seguindo suas fibras de distal a proximal com um dissector rombo. Sob esse ligamento, o TP foi isolado e identificado por meio da tração do ligamento fibular poplíteo.

Após a identificação do LCL e do TP, o LAL foi reconhecido e confirmado pelo tensionamento de suas fibras e visualização de seus sítios de inserção femoral e tibial. Na etapa final do experimento, foi realizada a secção cirúrgica transversal do LCA e as medidas internas foram registradas, assim como nas etapas anteriores do experimento. Estes dados foram coletados em um grupo denominado ligamento cruzado anterior com lesão, trato iliotibial com lesão e ligamento anterolateral com lesão (LCA L + TIT + LAL). O procedimento experimental foi o mesmo nos dois joelhos.

### Análise Estatística

As variáveis foram descritas como média e desvio padrão (DP). A comparação da amplitude de movimento entre os procedimentos foi feita por meio de análise de variância (ANOVA) para medidas repetidas, sendo complementada pelo teste post hoc de Bonferroni quando apropriado. O nível de significância adotado foi de 5% ( *p* < 0,05) e as análises foram realizadas com o *software* the IBM SPSS Statistics for Windows (IBM Corp.), versão 21.0. O teste *t* para amostras pareadas comparou os valores médios das medidas de rotação interna com a presença de lesões do LCA e a adição de secções do LAL. Nesta análise, o nível de significância adotado foi de 1% ( *p* < 0,001).

## Resultados

O LAL foi identificado como uma estrutura anatômica distinta em todos os 38 espécimes, mas somente após a retração de todas as camadas do TIT a partir de seu sítio de inserção tibial distal. Em todos os 38 joelhos, a inserção do LAL no menisco lateral pôde ser identificada anatomicamente. A manipulação do menisco lateral em todas as direções mostrou que as fibras de inserção do LAL seguiam a mesma direção do menisco lateral durante sua movimentação. A inserção do LAL na tíbia estava, em média, a meio caminho entre o ponto médio do tubérculo de Gerdy e a inserção do LCL na cabeça da fíbula.

A secção isolada do LCA induziu um aumento significativo (+55,6%, *p* < 0,001) na rotação interna do joelho a 90 graus de flexão em comparação ao joelho intacto.

Após a secção do LCA, a associação da liberação do TIT induziu um aumento adicional da rotação interna do joelho a 90 graus de flexão (+31,6%, *p* < 0,001) e um aumento muito significativo (+104%, *p* < 0,001) quando comparado ao joelho intacto.

Após a secção do LCA e do TIT, a liberação adicional do LAL induziu um aumento significativo na rotação interna do joelho a 90 graus de flexão (+27,8%, *p* < 0,001) e quando comparado ao joelho com LCA intacto (+162%, *p* < 0,001).

Observou-se aumento significativo na rotação interna do joelho à medida que as secções ligamentares eram realizadas. A secção específica do LCA levou a um aumento de 55,6% na rotação interna média do joelho em relação ao fêmur em comparação à média do grupo com LCA intacto.

A adição da liberação do TIT aumentou a rotação interna média do joelho em até 102%. Por fim, após a secção do LAL, a rotação interna média do joelho aumentou ainda mais, em até 162%.

Houve diferença significativa entre as medidas médias de rotação interna (21,13 ± 3,68) após a secção isolada do LCA em comparação à rotação interna média (35,57 ± 6,81) obtida após a secção do LAL ( Tabela 1 ). Logo, essa associação gerou um aumento de 68,3% na rotação interna quando comparada às medidas após a secção isolada do LCA ( Tabela 2 ).

**Tabela 1:** Diferenças de rotação após a secção seriada em 90 graus de flexão

Amplitude de movimento	LCA I	LCA L	LCA L + TIT	LCA L + TIT + LAL	*p* -value
	Média ± DP	Média ± DP	Média ± DP	Média ± DP	
Rotação interna do joelho direito	13,6 ± 3,9	21,0 ± 3,8	27,4 ± 5,0	36,5 ± 7,4	< 0,001

**Abreviações:** DP, desvio-padrão; LCA I, ligamento cruzado anterior intacto; LCA L, ligamento cruzado anterior com lesão, LCA L + TIT, ligamento cruzado anterior com lesão e TIT com lesão; LCA L + TIT + LAL, ligamento cruzado anterior com lesão, trato iliotibial com lesão e ligamento anterolateral com lesão.

**Nota:** Diferença estatística em 5% segundo o teste *post hoc* de Bonferroni.

**Tabela 2:** Aumento da rotação interna do joelho após secções ligamentares sequenciais

Condição	Média ± DP		Deslocamento (%)
LCA I	13,57 ± 4,195		0
LCA L	21,13 ± 3,684		55,6
LCA L + TIT	27,81 ± 4,543		104
LCA L + TIT + LAL	35,57 ± 6,812		162

**Abreviações:** DP, desvio-padrão; LCA I, ligamento cruzado anterior intacto; LCA L, ligamento cruzado anterior com lesão, LCA L + TIT, ligamento cruzado anterior com lesão e TIT com lesão; LCA L + TIT + LAL, ligamento cruzado anterior com lesão, trato iliotibial com lesão e ligamento anterolateral com lesão.

**Nota:** Comparação de médias, desvios-padrão (DP) e porcentagem de deslocamento anterolateral da rotação interna entre os grupos.

O grupo LCA L + TIT + LAL apresentou rotação interna média do joelho significativamente maior ( *p* < 0,001) do que grupo LCAL (deficiência isolada do LCA).

Este experimento mostrou um aumento pronunciado na rotação interna do joelho após a lesão do LAL em joelhos com deficiência do LCA; sendo relacionado à liberação do LAL.

## Discussão

As secções isoladas do LCA provocaram um aumento significativo da rotação interna do joelho a 90 graus de flexão em comparação a joelhos intacto. A liberação associada do TIT em joelhos com insuficiência do LCA deficiente induziu mais um aumento significativo na rotação interna do joelho a 90 graus de flexão em comparação a joelhos LCA intacto, destacando sua relevância. A secção adicional do LAL aumentou significativamente a rotação interna do joelho.

Nosso estudo demonstrou que há um aumento significativo na rotação interna do joelho nos casos de insuficiência do LCA e do LAL e secção do TIT, sugerindo que não há uma estrutura específica que controle a rotação do joelho. No entanto, nossos resultados demonstram que a insuficiência do LAL aumenta a instabilidade rotacional em comparação às secções do LCA e do TIT. Sabe-se que as estruturas anterolaterais são importantes restrições à rotação interna do joelho, 4 10 11 atuando em sinergia com o LCA. 12

O ruído produzido no teste de *pivot-shift* parece ter uma correlação fraca com a lesão do LCA e dessas estruturas. 13 Monaco et al. 13 foram os primeiros a levantar a hipótese da relevância do LAL e outras estruturas anterolaterais do joelho. Parsons et al. 14 demonstraram que a contribuição do LAL aumenta significativamente com a maior flexão do joelho, enquanto o LCA reduz sua contribuição também de maneira significativa. A contribuição do LAL supera a do LCA após 30 graus de flexão do joelho. Os autores concluíram que o LAL é um importante estabilizador da rotação interna do joelho após 35 graus de flexão. Sonnery-Cottet et al. 9 confirmaram e enfatizaram a participação do LAL no controle da rotação interna do joelho dentre as estruturas anterolaterais.

A ruptura do LCA desloca o eixo de rotação em direção ao compartimento medial do joelho, aumentando não só a translação anterior da tíbia sobre o fêmur, mas também a rotação interna do compartimento lateral. 10 Assim, um aumento significativo no recrutamento da estrutura anterolateral é necessário para restringir tal movimento. Os achados do nosso estudo confirmam esse fato, como mostra a Tabela 1 . A insuficiência do controle rotatório pós-operatório, observado após a reconstrução clássica do LCA, pode ser causada pela modificação do centro de rotação do joelho, mas também pela associação com lesão das estruturas. 15

Uma publicação recente relatou resultados promissores da reconstrução do LCA e do LAL em termos de desfechos clínicos e controle rotacional com acompanhamento superior a 2 anos. 16 Curiosamente, nessa série, a taxa de lesão do LCA contralateral (6,6%) foi semelhante à descrita na literatura, mas a taxa de ruptura do enxerto do LCA associada à reconstrução do LAL (1,1%) foi menor do que as já publicadas, 17 18 19 demonstrando que tal associação pode ser extremamente benéfica em alguns casos.

Em relação às restrições primárias e secundárias, a insuficiência de restrição primária recruta estruturas secundárias para resistir às forças externas e estabilizar o movimento articular. Em um estudo sobre o LAL, Dodds et al. 20 demonstraram que a rotação interna da tíbia em relação ao fêmur aumenta a distância entre suas inserções nesse ligamento, levando a estreitamento. Os autores relataram que a persistência da instabilidade rotacional após a reconstrução do LCA pode ser provocada por falha na correção da insuficiência das estruturas anterolaterais. Nosso estudo demonstrou que, em joelhos com secção do LCA (21,13 ± 4,19), ou seja, com instabilidade rotacional anterolateral simples, a liberação adicional do LAL (35,57 ± 6,81) aumentou significativamente a rotação interna ( *p* < 0,001).

Este estudo apresenta algumas limitações. A sequência de secções pode ter super- ou subestimado a estabilidade individual de cada componente devido às interações entre essas estruturas anatômicas, que não puderam ser avaliadas apenas pela técnica de dissecção. O método de medição não é eletronicamente preciso para coleta de dados (como a navegação). No entanto, todas as medidas foram submetidas a um protocolo de execução rígido e sempre realizadas pelo mesmo pesquisador. Não isolamos ou testamos as fibras de Kaplan do TIT, nem testamos as rotações com diferentes torques de força. Portanto, nossos resultados podem depender das cargas aplicadas a essas estruturas.

Apesar da dissecção meticulosa do TIT, o LAL pode ter sido sofrido lesão, o que modificaria os resultados. Não testamos a secção isolada porque isso não ocorre clinicamente. A associação entre a liberação do LAL e do LCA não foi avaliada por ainda não ser possível. Isto se deve à impossibilidade de isolamento do LAL para realizar sua secção sem antes retrair o TIT, seguindo rigorosamente a técnica de dissecção, como bem discutido por Sonnery-Cottet et al. 9

## Conclusão

A lesão do LCA aumenta a rotação interna do joelho. Há uma correlação entre a lesão do LCA e do LAL, causando um aumento pronunciado da rotação interna em comparação ao joelho não lesionado (acima de 100%). Portanto, a presença de rotação interna pronunciada do joelho é um sinal clínico de lesão associada dessas estruturas, o que pode auxiliar os cirurgiões no diagnóstico e orientar tratamentos mais adequados.
